# DNA Demethylation Upregulated Nrf2 Expression in Alzheimer’s Disease Cellular Model

**DOI:** 10.3389/fnagi.2015.00244

**Published:** 2016-01-05

**Authors:** Huimin Cao, Li Wang, Beibei Chen, Peng Zheng, Yi He, Yubin Ding, Yushuang Deng, Xi Lu, Xiuming Guo, Yuping Zhang, Yu Li, Gang Yu

**Affiliations:** ^1^Department of Neurology, The First Affiliated Hospital of Chongqing Medical University, Chongqing, China; ^2^Chongqing Key Laboratory of Neurology, The First Affiliated Hospital of Chongqing Medical University, Chongqing, China; ^3^Department of Bio-therapy and Hemato-oncology, Chongqing Cancer Institute, Chongqing, China; ^4^Department of Reproductive Biology, Chongqing Medical University, Chongqing, China; ^5^Department of Pathology, Chongqing Medical University, Chongqing, China; ^6^Institute of Neuroscience, Chongqing Medical University, Chongqing, China

**Keywords:** Alzheimer’s disease, DNA demethylation, Nrf2, NQO1, oxidative stress, cellular model

## Abstract

Nuclear factor erythroid 2-related factor 2 (Nrf2) is an important transcription factor in the defense against oxidative stress. Cumulative evidence has shown that oxidative stress plays a key role in the pathogenesis of Alzheimer’s disease (AD). Previous animal and clinical studies had observed decreased expression of Nrf2 in AD. However, the underlying regulation mechanisms of Nrf2 in AD remain unclear. Here, we used the DNA methyltransferases (Dnmts) inhibitor 5-aza-2′-deoxycytidine (5-Aza) to test whether Nrf2 expression was regulated by methylation in N2a cells characterizing by expressing human Swedish mutant amyloid precursor protein (N2a/APPswe). We found 5-Aza treatment increased Nrf2 at both messenger RNA and protein levels via downregulating the expression of Dnmts and DNA demethylation. In addition, 5-Aza-mediated upregulation of Nrf2 expression was concomitant with increased nuclear translocation of Nrf2 and higher expression of Nrf2 downstream target gene NAD(P)H:quinone oxidoreductas (NQO1). Our study showed that DNA demethylation promoted the Nrf2 cell signaling pathway, which may enhance the antioxidant system against AD development.

## Introduction

Alzheimer’s disease (AD) is a common neurodegenerative neurological disorder characterized pathologically by senile plaques, neurofibrillary tangles, and massive neuronal loss in the brain (Kumar et al., 2015). Numerous s1tudies have indicated that oxidative stress, an imbalance between free radicals and the antioxidant system, plays a significant role in the onset and progression of AD (Sultana et al., 2006; Zhao and Zhao, 2013). It has been reported that markers of oxidative stress were increased in the brains of AD patients (Williams et al., 2006). Evidence has also suggested that the activity of antioxidants is decreased in the early phase of AD (Cervellati et al., 2014). Because of the high concentration of polyunsaturated fatty acids and high oxygen consumptions in AD patients, those diseased brains were even more sensitive to oxidative stress (Farooqui and Horrocks, 1998). Thus, reducing oxidative damage in the brains could be a legitimate target for effective AD therapy.

Nuclear factor erythroid 2-related factor 2 (Nrf2) is a pivotal regulator of endogenous defense systems against oxidative stress, especially in the central nervous system (Yang et al., 2015). Nrf2 is mainly sequestered in the cytoplasm by Kelch-like ECH associated protein 1 (Keap1). In response to reactive oxygen species, Nrf2 could be translocated from the cytoplasm to the nucleus and subsequently binds with antioxidant response element (ARE). This process can promote expression of a variety of antioxidant genes, such as superoxide dismutase (SOD), catalase (CAT), hemeoxygenase-1 (HO-1), and NAD(P)H:quinone oxidoreductas (NQO1), which exert cytoprotective effects against oxidative stress (Li and Kong, 2009). Additionally, the nuclear translocation of Nrf2 is paralleled with its total protein and messenger RNA (mRNA) expression levels (Kanninen et al., 2009; Zhang et al., 2013). Despite that, previous animal and clinical studies had been consistently observing decreased Nrf2 level in hippocampus of AD (Ramsey et al., 2007; Tomobe et al., 2012; Farr et al., 2014). The underlying mechanisms of low expression of Nrf2 in AD patients remain largely unknown and need to be further investigated.

It is well established that DNA methylation affects gene expression (Momparler, 2005). DNA methylation blocks gene expression, whereas demethylation with 5-Aza activates gene expression (Lu et al., 2013). 5-Aza, the DNA methyltran ferases (Dnmts) inhibitor, could demethylate DNA and alter gene expression through downregulation of Dnmts or repression of Dnmts enzymatic activity (Juttermann et al., 1994; Qiu et al., 2002). 5-Aza is the first epigenetic drug that is approved by the Food and Drug Administration (FDA) for the treatment of myelodysplastic syndromes (MDS). Recently, Lu et al. (2013) found that the DNA methylation could regulate the Nrf2 level through modification of the first five CpG sites in prostate cancer.

Here, we used 5-Aza to investigate whether CpG methylation of the Nrf2 promoter could inhibit Nrf2 expression in the cellular model of AD. The aim of our study is to further explore if the expression of Nrf2 is regulated by DNA methylation and if upregulation of Nrf2 via DNA demethylation would promote the nuclear translocation of Nrf2 and the expression of its downstream gene NQO1.

## Materials and Methods

### Cell Culture and Drug Treatments

Mouse neuroblastoma N2a cell stably expressing human Swedish mutation APP (N2a/APPswe) gene were kindly provided by Dr. Huaxi Xu (Burnham Institute for Medical Research, La Jolla, CA, USA). N2a/APPswe cells were maintained in 50% Dulbecco’s modified Eagle’s medium (DMEM), 50% OPTI-MEM plus 5% fetal bovine serum (Gibico, Carlsbad, CA, USA) in the presence of 150 μg/ml G418 and plated at a density of 2 × 10^7^ in 75 cm^2^ cell culture flasks. 5-Aza-2′-deoxycytidine (5-Aza) (Sigma, USA) was dissolved in DMSO according to the manufacturer’s protocol. Cells were seeded for 24 h, then treated with 0.1% DMSO, 3 μM 5-Aza, and 5 μM 5-Aza for 72 h, maintained in a humidified incubator with 5% CO_2_ at 37°C. Cells of each group from three independent flasks were harvested for DNA, RNA, and protein analyses outlined below within each experimental procedure.

### Total RNA Extraction and Quantitative Real-Time Polymerase Chain Reaction

Total RNA was extracted from the cultured cells using the Trizol (Takara Bio, Japan) according to the manufacturer’s protocol. Then, first-strand cDNA was synthesized from total RNA by reverse transcription (RT) using PrimeScript RT kit (TaKaRa, Japan), real-time PCR was performed by Thermal Cycler Dice Real Time System (Thermo, USA) using SYBR PrimeScript PCR kit (Takara Bio, Japan). Quantitative real-time polymerase chain reaction (qPCR) was performed by an Eppendorf Mastercycler nexus instrument (Eppendorf, Germany) to quantify mRNA expression levels of Nrf2 and NQO1. The following sequences for the Nrf2 and NQO1 primers were used: Nrf2 forward (5′-GTGCTCCTATGCGTGAATCC-3′) and reverse (5′-GCGGCTTGAATGTTTGTCTT-3′), NQO1 forward (5′-GTTTCTGTGGCTTCCAGGTC-3′) and reverse (5′-CGTTTCTTCCATCCTTCCAG-3′). β-Actin was used as an internal control with sense (5′-GAGACCTTCAACACCCCAGC-3′) and antisense (5′-ATGTCACGCACGATTTCCC-3′) primers. Normalized values for specific gene mRNA expression were calculated as 2^−ΔΔCT^ method.

### Preparation of Protein Lyses and Western Blotting

N2a/APPswe cells were treated with indicated concentrations of 5-Aza. After the 72 h of treatment, cells were harvested using RIPA buffer supplemented with a protease inhibitor cocktail (Sigma, St. Louis, MO, USA) to obtain whole protein lysate. In the case of nuclear protein extraction, the Nuclear and Cytoplasmic Protein Extraction Kit (BestBio, China) was used according to the manufacturer’s instructions. The protein concentrations of the cleared lysates were determined using the bicinchoninic acid (BCA) method (Pierce, Rockford, IL, USA). Equal quantities of protein (50 μg per lane) were electrophoresed on SDS-PAGE (Bio-Rad, CA, USA) under reducing conditions and then electrophoretically transferred onto the polyvinylidene difluoride (PVDF) membranes (Millipore, USA) followed by blocking with 5% BSA for 1.5 h. The PVDF membranes were incubated overnight at 4°C probed with primary antibodies: Nrf2 (Abcam, USA; 1:1,000), NOQ1 (Genetex, USA; 1:1,000), Dnmt1, Dnmt3a, and Dnmt3b (Santa Cruz, CA, USA; all 1:400), β-actin (Santa Cruz, CA, USA; 1:3,000), and Lamin B (Beijing TDY Biotech, China; 1:3,000). β-Actin or Lamin B was used as a loading control. After washing with TBST, membranes were then incubated with horseradish peroxidase-conjugated secondary antibody (BOSTER, Wuhan, China, 1:2,000). The immunoreactive protein bands were detected with a Chemiluminescence Luminal reagent (Keygen, China) and the intensity of the bands was analyzed with Quantity One (Bio-Rad, USA).

### Immunofluorescence Staining

N2a/APPswe cells treated with or without 5-Aza were fixed with 4% paraformaldehyde for 15 min at room temperature on the slides and followed by a rinse with phosphate-buffered saline (PBS) three times for 15 min. After washing, cells were incubated with 0.3% Triton for 15 min at room temperature and then blocked with 5% BSA for 30 min. The cells were incubated with Nrf2 antibody at 1:100 (Abcam, USA) overnight at 4°C. Next day the cells were washed three times in PBS for 15 min. Then, the cells were incubated with goat anti-rabbit conjugated to DyLight 549 secondary antibody at 1:200 (Abbkine, USA) for 30 min at 37°C. Later, the cells were washed three times in PBS for another 15 min and then stained with DAPI (Beyotime, Dalian, China) for 3 min at room temperature. Finally, cells were washed in PBS for 15 min and observed with a Confocal Laser-scanning Microscope (A1R, Nikon, Japan).

### Bisulfite-Sequencing PCR

Genomic DNA was isolated from 0.1% DMSO and 5 μM 5-Aza treated N2a/APPswe cells using the DNeasy Tissue Kit (QIAGEN, Valencia, CA, USA). The bisulfite conversion was carried out by Methylamp DNA Modification Kit (Epigentek, Brooklyn, NY, USA) with 2 μg of genomic DNA by the manufacturer’s instructions. The converted DNA was amplified by PCR using Platinum BluePCR SuperMix (Invitrogen, Grand Island, NY, USA) and primers that amplify the first five sites located between −1226 and −1175 of the murine Nrf2 gene with the translational start site defined as +1. The sequences of the primers were as follows: 5′-GAGTTATTTTAAGTATTTTAGATATTTTG-3′ (sense) and 5′-ATACTCAAACACCTCTACCCC-3′ (antisense). PCR products were separated on agarose gels that were excised for the DNA fragment containing the first five CpG sites for being cloned into the pUC18-T vector (Biodee, Beijing, China). Plasmid DNA from at least 10 colonies per each group was purified using the QIAquick PCR purification kit (QIAGEN, Valencia, CA, USA) and sequenced by Sangon Biotechnology.

### Statistical Analysis

All results are expressed as means ± SD. *Post hoc* tests following the one-way ANOVA analyses were used to determine the differences between multiple groups. The chi-square test was used to analyze the first five CpG site methylation rates of Nrf2 gene. A value of *P* < 0.05 was considered statistically significant.

## Results

### 5-Aza Increases mRNA and Protein Expression of Nrf2 in N2a/APPswe Cells

**Figure 1:**
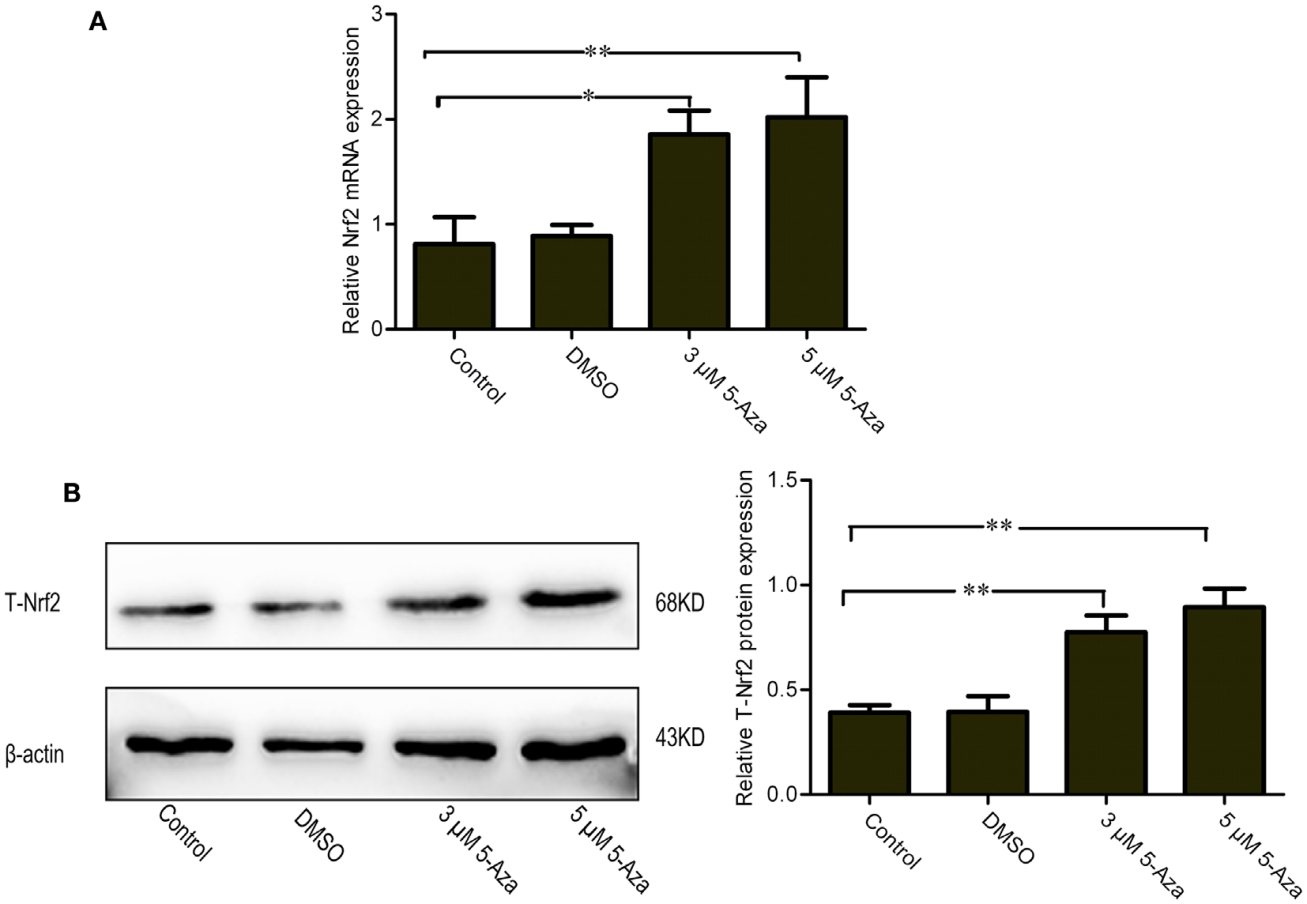
**Upregulation of mRNA and protein levels of Nrf2 by 5-Aza treatment in N2a/APPswe cells**. N2a/APPswe cells were treated with 0 μM 5-Aza (control), 0.1% DMSO, 5-Aza (3 and 5 μM) for 72 h. The expressions of Nrf2 and β-actin mRNA were measured by Q-PCR using total RNA from N2a/APPswe cells. β-Actin was assessed as a loading control **(A)**. The protein expressions of total Nrf2 (T-Nrf2) and β-actin were measured by Western blot analysis using lysates from N2a/APPswe, normalized to β-actin expression **(B)**. All data were represented as a mean ± SD of three independent experiments. *P < 0.05 and **P < 0.01.

To investigate the effect of DNA methylation against Nrf2 expression in N2a/APPswe cells, the cells were treated with 0 μM 5-Aza (control), 0.1% DMSO, 5-Aza (3 and 5 μM) for 72 h. The relatively low Nrf2 mRNA and protein levels were found in control group. By contrast, the mRNA and protein of Nrf2 were significantly increased by 5-Aza treatment (Figures 1A,B). These findings suggest that 5-Aza can increase the expression of Nrf2.

### 5-Aza Suppresses Dnmts Protein Expression

**Figure 2:**
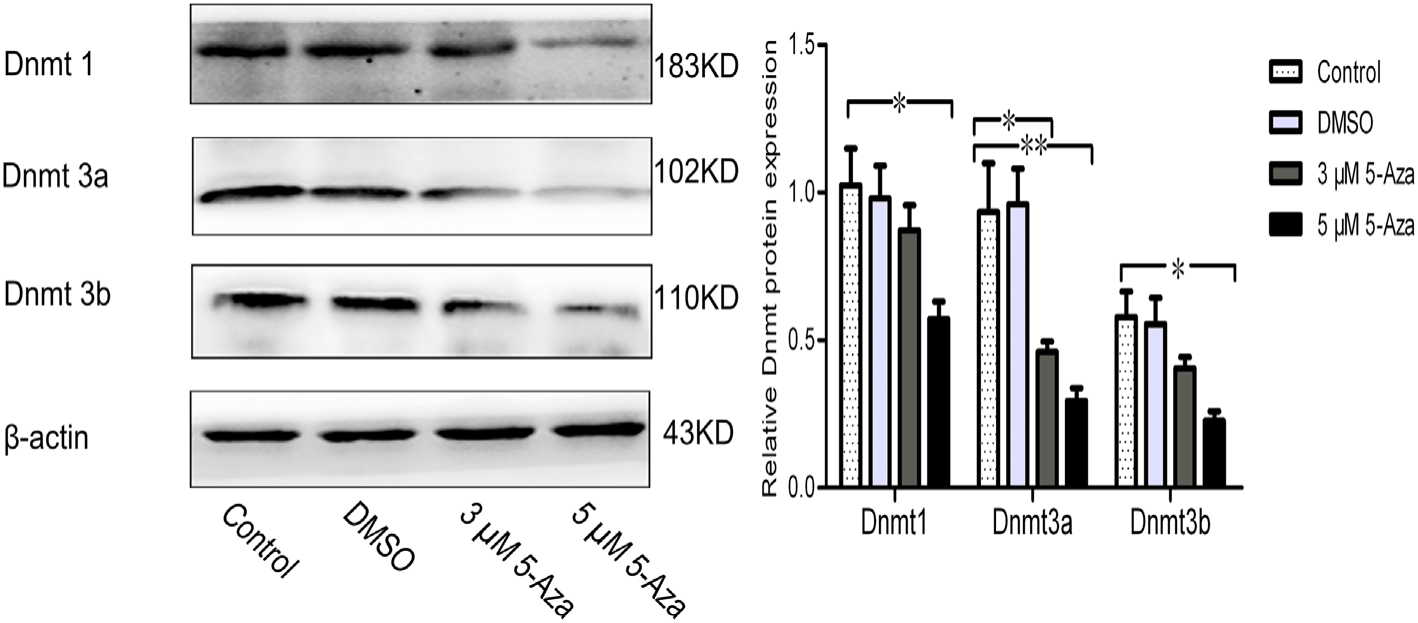
**5-Aza suppresses Dnmts protein expression**. N2a/APPswe cells were treated with 0 μM 5-Aza (control), 0.1% DMSO, 5-Aza (3 and 5 μM) for 72 h. The protein expressions of Dnmt1, Dnmt3a, Dnmt3b, and β-actin were measured by Western blot. β-Actin was assessed as a loading control. All data were represented as a mean ± SD of three independent experiments. *P < 0.05 and **P < 0.01.

Because DNA methylation occurs at the 5′ position of the cytosine residue within CpG dinucleotides through the addition of a methyl group by DNA methyltransferases (Dnmts), including Dnmt1, Dnmt3a, and Dnmt3b in mammals (Subramaniam et al., 2014). Our results showed that the Dmnts expression has been reduced by application of 5-Aza in N2a/APPswe cells (Figure 2), indicating that 5-Aza can deprive of Dnmts activity, leading to the lower DNA methylation.

### The First Five CpG Sites in the Nrf2 Promoter Are Specifically Demethylated by 5-Aza Treatment

**Figure 3:**
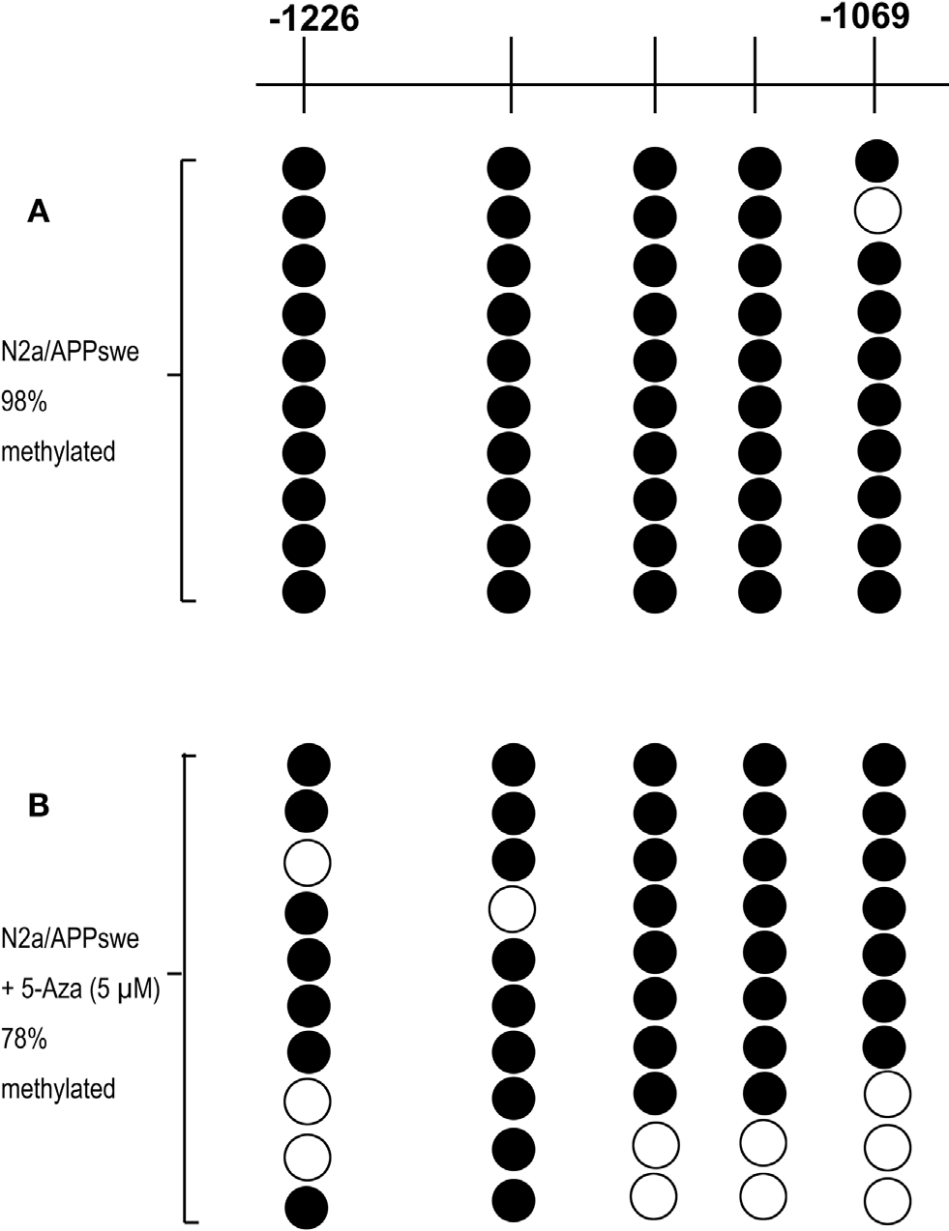
**The first five CpG sites are specifically demethylated by 5-Aza treatment**. N2a/APPswe cells were treated with 0.1% DMSO, 5 μM 5-Aza for 72 h. The methylation patterns of the first five CpG sites of promoter Nrf2 gene in N2a/APPswe cells were performed using bisulfite genomic sequencing (BGS) as described in Section “Materials and Methods.” Black circles indicate methylated CpGs, and open circles indicate unmethylated CpGs. The first five CpGs were hypermethylated in N2a/APPswe cells, which were treated with 0.1% DMSO (98% methylated) **(A)** and cells treated with 5 μM 5-Aza for 72 h (78% methylated) (chi-square test, *P* < 0.05) **(B)**.

One hundred fifty CpG sites ranging from −1226 to +1240 were identified on the murine of Nrf2 genomic sequence by sequence analysis. The CpG island includes the murine Nrf2 promoter, the first exon, and part of the first intron. Yu et al. (2010) proposed that the first five CpG sites of Nrf2 (−1226 to −1175) play a critical role in methylation-dependent suppression of Nrf2 promoter activity that designates the initiation site of translation as +1. To identify whether the first five CpG sites responsible for Nrf2 expression in N2a/APPswe cells, bisulfite sequencing was performed on extracted DNA from N2a/APPswe cells that were incubated with or without 5-Aza treatment to quantitatively analyze the methylation levels of first five CpG sites in Nrf2 gene. Ten clones were selected to analyze for each CpG site. We found that 49 out of 50 (98%) CpG sites were methylated in basal N2a/APPswe cells without 5-Aza treatment (Figure 3A). Five micromolar of 5-Aza was then selected to treat N2a/APPswe cells with regard to its significant upregulation of Nrf2 expression (Figures 1A,B). Results have shown that Nrf2 promoter methylation decreased to 39 out of 50 (78%) in the first five CpG sites after treated with 5 μM 5-Aza (chi-square test, *P* < 0.05) (Figure 3B), indicating that the first five CpG sites of Nrf2 promoter have been demethylated by 5-Aza treatment.

### 5-Aza Promotes the Nuclear Translocation of Nrf2 and Increases mRNA and Protein Expression Levels of Nrf2 Downstream Gene

**Figure 4:**
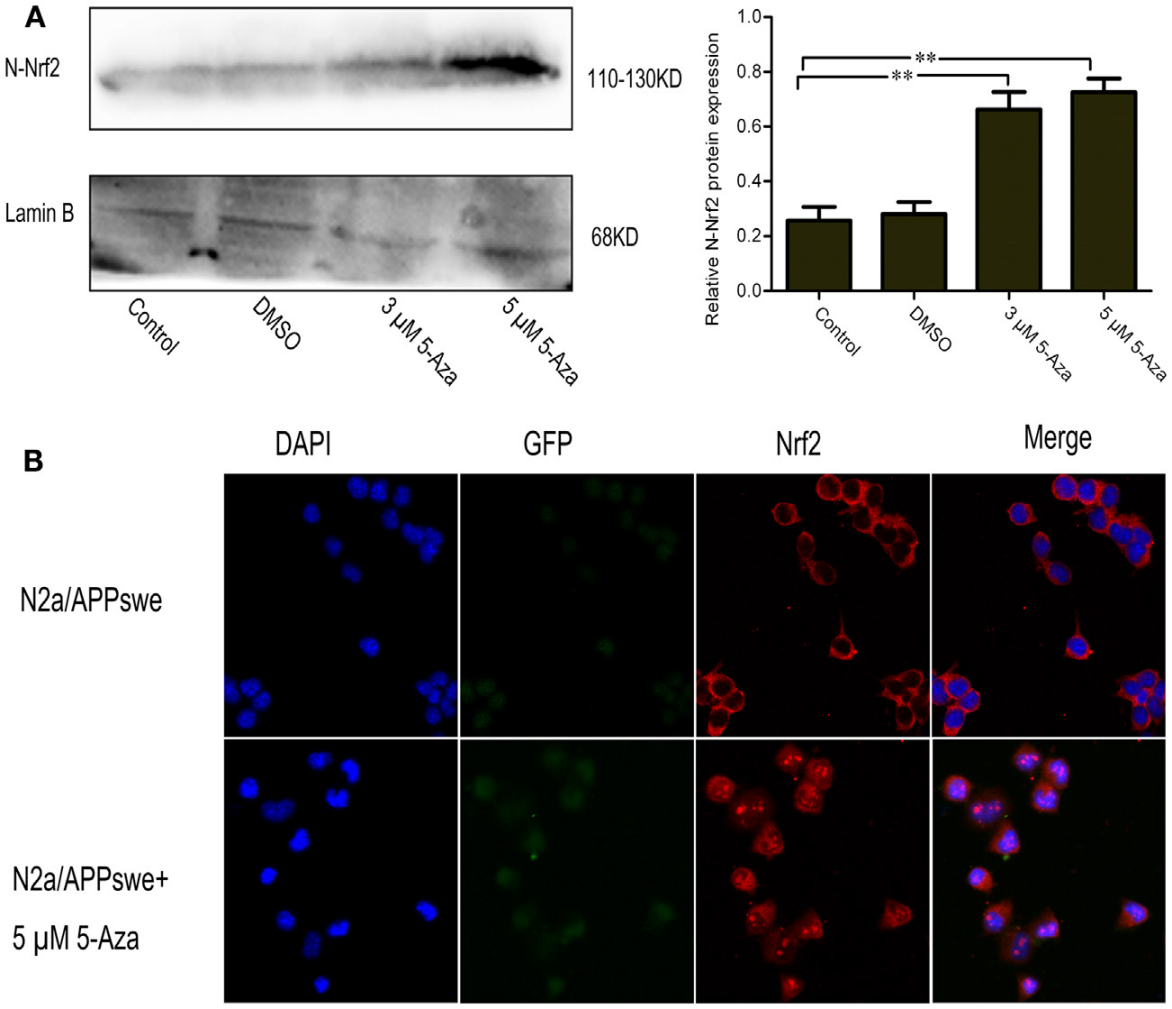
**5-Aza promotes the nuclear translocation of Nrf2 in N2a/APPswe cells**. N2a/APPswe cells were treated with 0 μM 5-Aza (control), 0.1% DMSO, 5-Aza (3 and 5 μM) for 72 h. The protein expressions of N-Nrf2 (nucleus protein of Nrf2) and Lamin B were measured by Western blot using nuclear protein from N2a/APPswe cells. Lamin B was assessed as a loading control **(A)**. Immunofluorescence staining was used to observe the distribution of Nrf2 **(B)**. N2a/APPswe cells were treated with 5 μM 5-Aza for 72 h and labeled with anti-Nrf2 antibody (red) and DAPI (blue), APPswe were labeled with GFP (green). Scale bar 50 μm. All data were represented as a mean ± SD of three independent experiments. **P < 0.01.

**Figure 5:**
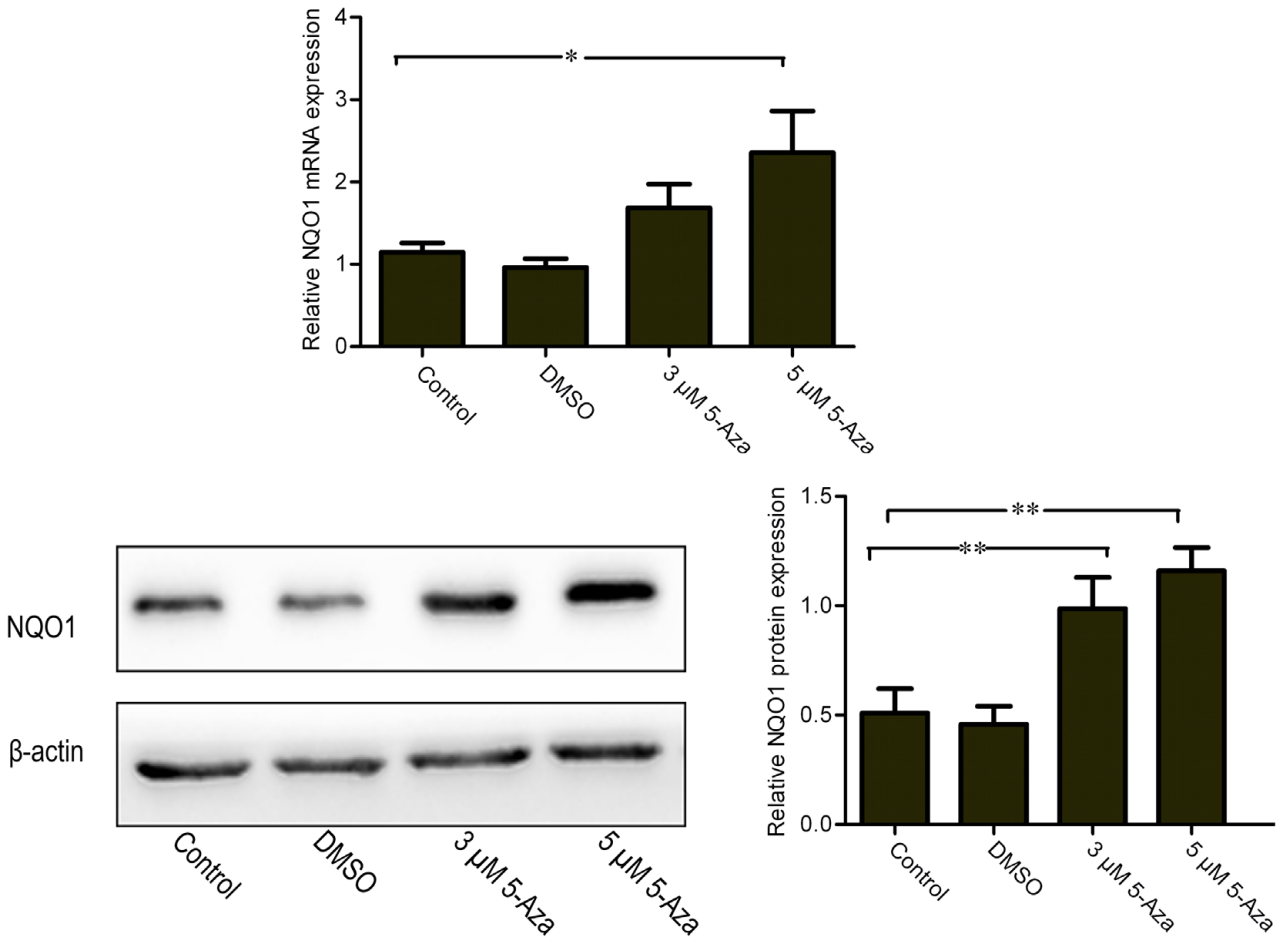
**5-Aza increases mRNA and protein expression levels of Nrf2 downstream gene NQO1**. N2a/APPswe cells were treated with 0 μM 5-Aza (control), 0.1% DMSO, 5-Aza (3 and 5 μM) for 72 h. The protein expressions of NQO1 and β-actin were measured by Western blot analysis using total protein from N2a/APPswe. β-Actin was assessed as a loading control. All data were represented as a mean ± SD of three independent experiments. *P < 0.05 and **P < 0.01.

To determine whether the upregulation of Nrf2 via DNA demethylation could promote the activation of Nrf2, the nuclear translocation of Nrf2 induced by 5-Aza was further assessed. We found that 5 μM of 5-Aza can significantly increase the nuclear translocation of the Nrf2 protein in N2a/APPswe cells (Figure 4A). This finding was further confirmed by immunofluorescence staining (Figure 4B). To determine how upregulation of Nrf2 influences the oxidative stress status, one of the Nrf2 downstream target genes, NQO1, was selected for quantification. We found that the mRNA and protein expressions of NQO1 were significantly increased in 5-Aza treated group compared with that in control group in N2a/APPswe cells (Figure 5).

## Discussion

Cumulative evidence has demonstrated that oxidative stress is inextricably linked with several major pathological processes in AD (Sheng et al., 2009; Clausen et al., 2012; Porcellotti et al., 2015). Nrf2, a pivotal regulator of endogenous defense systems against oxidative stress, is decreased in AD brain tissue (Kanninen et al., 2009). In previous studies, Yu et al. (2010) demonstrated DNA methylation in the first five CpG sites of Nrf2 gene silenced its transcriptional activity. Here, by using the N2a/APPswe cell lines characterized by oxidative stress injury, we found Nrf2 expression is regulated by DNA methylation. 5-Aza treatment can lead to the increase in the mRNA and protein expressions of the Nrf2 gene. Furthermore, 5-Aza specifically decreased the expression of Dnmt1, Dnmt3a, and Dnmt3b and demethylated the first five CpG sites in the promoter of Nrf2. Our results suggest that the first five CpG sites in the promoter may play an important role in Nrf2 expression via an epigenetic regulation of DNA methylation in AD cellular model. In addition, we found 5-Aza can promote the nuclear translocation of Nrf2 and increase the mRNA and protein expressions of NQO1 in N2a/APPswe cells. Our findings strongly suggest that DNA demethylation may be an effective pathway to reactivate the Nrf2 cell signaling in AD cellular model.

The methylation of CpG dinucleotides at the 5′ position on the pyrimidine ring to form 5-methylcytosine (5-mC) disrupts the cellular transcriptional machinery and results in gene silencing. In contrast, DNA hypomethylation is associated with upregulation of gene expression (Bhutani et al., 2011). Previous studies demonstrated that aberrant DNA methylation of genes was associated with initiation and progression of AD (Adwan and Zawia, 2013). In a recent study, Fleming et al. (2012) found that DNA hypomethylation may account for the upregulation of three well-established AD-related genes (APP, BACE1, and PS1). DNA hypermethylation of CpG islands in the promoter region of genes is associated with transcriptional silencing (Subramaniam et al., 2014). Our results showed that, a lower Nrf2 level and hypermethylation of the first five CpG sites were observed in untreated N2a/APPswe cells, indicating that hypermethylation was closely related to the low Nrf2 gene expression. Demethylation of Nrf2 gene promoter CpG sites could restore the gene expression.

In our study, we investigated whether 5-Aza, a Dnmts inhibitor, could reactivate the expression of Nrf2 gene in N2a/APPswe cells. The results showed that 5-Aza treatment can restore the expression of this gene. We then tested the expression of Dnmt1, Dnmt3a, and Dnmt3b that all mediate the effects of 5-Aza in mammals (Liao et al., 2015) and found the decreased expression of Dnmts in 5-Aza treated N2a/APPswe cells. Our results corroborate with previous findings that 5-Aza activated gene expression in a methylation-dependent manner (Schmelz et al., 2005). Using bisulfite sequencing, we found that the first five CpGs in Nrf2 promoter were hypermethylated in solvent control group but not in 5-Aza treated group (98 vs. 78%). Combined with a previous study that methylation of the first five CpGs can significantly suppress the transcriptional activity of Nrf2 in mouse (Yu et al., 2010), we propose that 5-Aza indeed affects expression of Nrf2 through repression of Dnmts expression in N2a/APPswe cells, demonstrating that DNA methylation can regulate the expression of Nrf2 in AD cellular model.

In the present work, we observed that Nrf2 could not be translocated to the nucleus or was less antigenically available in AD neuronal nuclei. This finding is in accordance with a report that Nrf2 does not respond to the oxidative stress in AD (Ramsey et al., 2007). Translocation of Nrf2 into the nucleus is also regulated by phosphorylation via several kinases including phosphoinositol-3 kinase (PI3K), extracellular signal-regulated protein kinase (ERK), protein kinase C (PKC), and pancreas-enriched kinase (PERK) (Bryan et al., 2013). Interestingly, in this study, we found that increasing both Nrf2 mRNA and protein levels by DNA demethylation resulted in remarkable increases of nucleus protein of Nrf2 (N-Nrf2). In addition, the 5-Aza treated N2a/APPswe cells also showed more nuclear immunofluorescence than that in solvent control group using immunofluorescence staining. Vargas et al. (2005) also reported that increasing both Nrf2 mRNA and protein levels by fibroblast growth factor family could contribute to activation of Nrf2 and induction of ARE-driven genes. These data indicate that the activity of Nrf2 is related to its total protein and mRNA expression levels. Taken together, these findings suggest that the regulation of Nrf2 gene has multiple pathways.

NQO1, an antioxidant enzyme, is one of the target genes of Nrf2 and plays a key role in maintenance of cellular redox state by reduction of various quinones and by prevention of reactive oxygen species accumulation (Zhou and Seeley, 2014). Here, we found that 5-Aza can increase the mRNA and protein expression levels of NQO1, which correlates with the increased total expression and nuclear translocation of Nrf2 in N2a/APPswe cells. These findings are consistent with the results that intrahippocampal injection of a lentiviral vector expressing Nrf2 can improve spatial learning in a mouse model of AD by reducing oxidative stress (Kanninen et al., 2009).

Alzheimer’s disease is driven by two processes, extracellular deposition of beta amyloid (Aβ) and intracellular accumulation of tau protein. Recent studies suggest that the activation of the Nrf2 pathway may have the effect of anti-amyloidogenic and reduces the levels of phosphorylated tau (Jo et al., 2014; Joshi et al., 2015). Our current studies proposed a possible pathway for AD pathogenesis and may provide an important clue for prevention and treatment of AD targeting on Nrf2 gene demethylation. Further studies to downregulate the Dnmts and determinate its phenocopy *in vitro* is required.

In conclusion, to the best of our knowledge, the present study for the first time to demonstrate that DNA methylation at the first five CpG sites in the promoter of Nrf2 is associated with the regulation of Nrf2 expression as well as its cell signaling pathway in AD cellular model. However, our findings are limited to the nature of the *in vitro* system, and the results will need to be validated by *in vivo* studies.

## Author Contributions

HC, LW, and BC have contributed equally to this work. Conceived and designed the experiments: GY, HC, BC, YD, XL, and LW. Performed the experiments: HC, BC, and LW. Analyzed the data: GY, HC, BC, and LW. Contributed reagents/materials/analysis tools: HC, BC, XG, YZ, and YL. Wrote the paper: HC, BC, PZ, YD, and XL.

## Conflict of Interest Statement

The authors declare that the research was conducted in the absence of any commercial or financial relationships that could be construed as a potential conflict of interest.
